# Chronic Hyperinsulinaemic Hypoglycaemia in Rats Is Accompanied by Increased Body Weight, Hyperleptinaemia, and Decreased Neuronal Glucose Transporter Levels in the Brain

**DOI:** 10.1155/2017/7861236

**Published:** 2017-03-21

**Authors:** Vivi F. H. Jensen, Anne-Marie Mølck, Melissa Chapman, Lene Alifrangis, Lene Andersen, Jens Lykkesfeldt, Ingrid B. Bøgh

**Affiliations:** ^1^Department of Veterinary Disease Biology, Section for Experimental Animal Models, Faculty of Health and Medical Sciences, University of Copenhagen, Copenhagen, Denmark; ^2^Department of Toxicology, Safety Pharm and Pathology, Novo Nordisk A/S, Maaloev, Denmark; ^3^Division of Toxicology, Envigo, Eye, Suffolk, UK; ^4^Department of Development DMPK, Novo Nordisk A/S, Maaloev, Denmark; ^5^Department of Development Bioanalysis, Novo Nordisk A/S, Maaloev, Denmark

## Abstract

The brain is vulnerable to hypoglycaemia due to a continuous need of energy substrates to meet its high metabolic demands. Studies have shown that severe acute insulin-induced hypoglycaemia results in oxidative stress in the rat brain, when neuroglycopenia cannot be evaded despite increased levels of cerebral glucose transporters. Compensatory measures in the brain during chronic insulin-induced hypoglycaemia are less well understood. The present study investigated how the brain of nondiabetic rats copes with chronic insulin-induced hypoglycaemia for up to eight weeks. Brain level of different substrate transporters and redox homeostasis was evaluated. Hyperinsulinaemia for 8 weeks consistently lowered blood glucose levels by 30–50% (4–6 mM versus 7–9 mM in controls). The animals had increased food consumption, body weights, and hyperleptinaemia. During infusion, protein levels of the brain neuronal glucose transporter were decreased, whereas levels of lipid peroxidation products were unchanged. Discontinued infusion was followed by transient systemic hyperglycaemia and decreased food consumption and body weight. After 4 weeks, plasma levels of lipid peroxidation products were increased, possibly as a consequence of hyperglycaemia-induced oxidative stress. The present data suggests that chronic moderate hyperinsulinaemic hypoglycaemia causes increased body weight and hyperleptinaemia. This is accompanied by decreased neuronal glucose transporter levels, which may be leptin-induced.

## 1. Introduction

Acute hypoglycaemia is a common complication in type 1 as well as in type 2 diabetic patients during intensive insulin therapy [1–3]. Frequent episodes are likely to weaken the normal counterregulatory responses and patient awareness of hypoglycaemia and may thus over time increase the incidence and severity of such episodes [4–6]. Due to the high metabolic activity of neurons, requiring a continuous supply of nutrients such as glucose or glucose-derived lactate, the brain is vulnerable to acute severe hypoglycaemia [7], which may lead to coma and neuronal necrosis [7–10].

Several studies have investigated the effect of acute insulin-induced hypoglycaemia in rats with blood glucose levels of 2-3 mmol/l or even below 2 mmol/l, that is, levels that may induce isoelectric EEG and coma [7, 8, 11–14]. Acute severe insulin-induced hypoglycaemia (<2.3 mM, ≤5 days) causes an adaptive increase in mRNA and protein levels of neuronal and blood-brain barrier (BBB) glucose transporters most likely to ensure adequate glucose supply to neurons [7, 11, 12, 15]. Indeed, insufficient cerebral glucose influx during hypoglycaemia has detrimental consequences leading to oxidative stress and regional neuronal death [7, 9, 16–18]. Thus, increased lipid oxidation can occur in the brain of rats and mice already after a single episode of insulin-induced hypoglycaemia if the hypoglycaemia is severe enough [17–19].

In contrast, effects of chronic (≥2 weeks) insulin-induced hypoglycaemia on cerebral glucose transporter levels or extent of oxidative damage to brain lipids have only been sparingly investigated [7]. The brain may be better equipped for prolonged but moderate lowering of blood glucose levels in contrast to acute or recurrent severe hypoglycaemia, which usually represents iatrogenic low blood glucose levels far below fasting levels, approaching a coma-inducing hypoglycaemic level. It is not clear if the brain is able to adapt to restricted glucose availability during extended periods and prevent neuroglycopenia or if cerebral damage due to oxidation of brain lipids will develop over time. Oxidative damage to brain lipids seems to be mediated through a reduction in antioxidant enzymes in the mitochondria combined with an increased production of reactive oxygen species in neurons [7, 16–20]. Furthermore, hypoglycaemia-induced oxidative damage to brain lipids seems to precede neuronal death [7, 19], suggesting that increased lipid peroxidation might be an early marker of hypoglycaemia-induced cerebral damage.

This prompted us to investigate how the brain from normoglycaemic healthy rats copes with long-term, persistent hyperinsulinaemic hypoglycaemia at a level corresponding to that achieved by fasting and using brain substrate transporter and lipid peroxidation product levels as indicators of adaptation and early damage, respectively. This should increase knowledge on regulation of brain transporters and increase the understanding of brain and body response to metabolic disturbances, which in this case consists of an induced paradoxical state of opposing metabolic signals of nutrient surplus (hyperinsulinaemia) and nutrient scarcity (hypoglycaemia), which would not otherwise occur together. Additionally, a period of recovery following eight weeks of hyperinsulinaemic hypoglycaemia was included to investigate reversibility of any changes induced.

## 2. Materials and Methods

### 2.1. Animals

Male and female Sprague-Dawley (Crl:CD (SD)) rats (*n* = 76/*sex*, 6-7 weeks old) were supplied by Charles River Limited (UK). The animals were acclimated to local environmental conditions (19–23°C, 40–70% humidity, 12 h light-dark cycle, lights on at 6:00 am) for at least five days and housed up to five animals/cage in solid-bottom polycarbonate cages with a stainless steel mesh lid and sawdust litter. They were offered a commercial diet (Rat and Mouse No. 1 Maintenance Diet, Special Diet Services, Essex, UK) and water ad libitum. The animals were randomised into four main groups stratified for body weight and sex (Table 1). To allow for infusion (see Study Design), the animals received surgical implantation of a vascular catheter inserted into the caudal vena cava through the right femoral vein as previously described [21]. Postsurgery animals were single-housed and allowed at least seven days of recovery before start of insulin infusion. Body weight was monitored daily for at least three days postsurgery or until the animals regained their presurgery body weights; hereafter, body weight and food consumption were monitored twice weekly. The animals were inspected visually for clinical signs of hypoglycaemia and function of the infusing system at least four times daily during the infusion phase. All procedures involving live animals were performed under the Project Licence authorized by the United Kingdom Secretary of State and according to EC Commission Directive 2004/10, OECD Principles and Good Laboratory Practice, and The Good Laboratory Practice (Codification Amendments Etc.) Regulations 2004 [22–24] as well as Envigo and Novo Nordisk A/S company policies on the care and use of laboratory animals.

### 2.2. Study Design

At infusion start (Day 1), male and female rats were approximately 8-9 weeks old, and they received either 28 (until Day 29) or 56 (until Day 57) full days of human insulin (HI), group HI-M and HI-F, or vehicle, group CTRL-M and CTRL-F, infusion or 56 full days of infusion (M: males, F: females). This was followed by either one (terminated on Day 2) or 28 (terminated on Day 29 after infusion-stop) full infusion-free days before termination. Doses are listed in Table 1. The aim was to approach the maximum tolerable dose of HI-infusion for up to 8 weeks in order to achieve a persistent and maximal pharmacodynamic effect. Consequently, the animals were closely monitored for clinical signs of hypoglycaemia, and doses were lowered, if the maximum tolerable dose was considered to be exceeded. Doses were chosen based on results from a previous 4-week HI-infusion study [21]. Females received lower doses than males as they are known to possess higher insulin sensitivity [25–27]. Flow rates (1.4 ml/kg/hour) were adjusted on a weekly basis according to individual body weight gain to keep doses constant in nmol/kg/day over the infusion period, whereas insulin concentration was kept constant for each dose level. Infusion formulation was renewed on a daily basis.

Animals with signs of hypoglycaemia (that is, tremor, underactivity/lethargy, piloerection, reduced body temperature, hunched posture, flat posture/collapsed, irregular breathing, and convulsions associated with low blood glucose values) were treated with glucose (p.o. or i.v.) immediately after recognition of clinical signs of hypoglycaemia. However, based on severe hypoglycaemic events leading to termination of the animals for welfare issues in three animals (two males and one female) within the first two weeks of the study, the dose levels were lowered in the male and female HI-infused groups (Table 1).

The animals were terminated after four or eight weeks of infusion on Day 29 (*n* = 9/group) or Day 57 (*n* = 10/group), respectively (Table 1). Infusion was ongoing until termination. After the end of infusion on Day 57, nineteen animals per group were kept on study and subjected to one (*n* = 9/group) or 28 (*n* = 10/group) full days of infusion-free recovery period, that is, to days 2 and 29 of recovery (Day R2 and R29). At termination, all the animals were subjected to a detailed necropsy. Right cerebrum hemisphere with right half of the cerebellum and left cerebrum hemisphere with left half of the cerebellum, respectively, were freeze-clamped in liquid nitrogen and stored at approximately −80°C. From the animals terminated on Day 29, 57, and R29, the right half was used for western blot analysis and the left half for quantification of brain tissue lipid oxidation.

### 2.3. Blood Sampling

Two types of sampling for blood glucose measurements were included: plasma (sublingual vein under isoflurane anaesthesia) for glucose profiling and single time-point measurements on selected days and whole blood (tail vein, no anaesthesia) to allow for continuous single time-point assessment of all the animals, which is not possible with the plasma measurements, as they require a larger blood volume as well as anaesthesia of the animal at each sampling.

Plasma was sampled for glucose profiling to monitor the decrease in blood glucose levels following the start of HI-infusion in the morning of Day 1 and on Day 25 and Day 53 (at 0.25, 0.5, 6, 12, or 24 h, relating to the start of infusion at 0 h on day 1); time points were determined based on the approximate half-life of HI in the rat of approximately 0.25 h [28], as well as on results from a previous study using the same animal model [21], and to confirm a persistent 24 h decrease as would be expected from continuous infusion. As this was the main purpose, only 2 animals/sex/group were sampled at each time point, to minimise stress (induced by handling and/or sampling) in the animals. Furthermore, the animals were sampled on the first day of the infusion-free recovery period (Day R1), at 0.25, 0.5, 6, 12, or 24 h after infusion-stop. In addition, blood samples for plasma glucose quantification were obtained at a single time point from all the animals on day 8 and before termination of the animals on Day 29, Day R2, and Day R29. Plasma glucose level was quantified as described previously [21]. On Day 1 and 53, blood was drawn also for plasma HI quantification at the same time points as for plasma glucose profiling measurements. Additional plasma samples for analysis of biomarkers of lipid oxidation were obtained at termination. Whole blood glucose levels were monitored with a snap blood glucose monitoring device (Accu-Chek Aviva, Cat/Type 05911974002, Roche Diagnostics, Burgess Hill, West Sussex, UK); all the animals were sampled twice weekly during the infusion period and once weekly during the infusion-free recovery period. The frequency of sampling was reduced if there was a scheduled plasma glucose profile within that week.

### 2.4. Insulin Formulations and Infusion System

The infusates used were recombinant HI stock solution formulated in a phosphate buffered vehicle (600 nmol/ml) and buffered vehicle (Novo Nordisk A/S, Maaloev, Denmark), diluted in dilution medium. Composition of buffered HI stock formulation, buffered vehicle, and dilution medium was as described previously [21]. For the infusion, external syringe infusion pumps and a vascular access harness connected to a tether kit were used as described previously [21].

### 2.5. Plasma HI Levels and Toxicokinetic Analysis

HI concentration was quantified in plasma by the use of a luminescent oxygen channelling immunoassay as described previously [21]. The lower limit of quantification (LLOQ) of the assay was 25 pmol/l. Samples were analysed in duplicate; results were reported as the mean of the two replicates.

For toxicokinetic analysis, composite mean profiles of plasma concentration versus time data from male and female rats were generated and subsequently treated as full profiles. The steady state concentrations (*C*_ss_) were calculated based on noncompartmental analysis. For a continuous i.v. infusion of HI, the plasma concentration of HI is per definition constant at all time points. For Day 1, the concentrations from 6 to 24 h represent *C*_ss_, as steady state is theoretically obtained after 5–7 half-lives (half-life of HI in rats is approximately 14 min [28]). Hence, on Day 1 from 6 to 24 h and for all time points on day 53, mean plasma concentrations for each animal and for each HI-infused group were calculated to yield individual and group mean *C*_ss_. Toxicokinetic calculations were performed in Phoenix™ WinNonlin® version 6.2, build 6.2.0.495 (Pharsight®, St. Louis, Missouri, USA).

### 2.6. Protein Extraction and Western Blotting

Approximately 35–45 mg of frozen pulverized brain tissue (right hemisphere with right half of the cerebellum) was homogenized in 250 *μ*l cold radio-immunoprecipitation assay (RIPA) buffer (50 nM Tris [pH 8.0], 150 mM NaCl, 1% Triton X-100, 0.5% sodium deoxycholate, and 0.1% sodium dodecyl sulfate) containing protease inhibitor cocktail diluted 1:100 (Sigma-Aldrich, St. Louis, MO, USA). The homogenate was centrifuged (10 min, 15300 ×g, 4°C) and the supernatant transferred to fresh Eppendorf tubes and frozen immediately at −20°C. Protein concentrations were determined in triplicate by a bicinchoninic acid assay (BCA Protein Assay Kit, Merck Life Science A/S, Hellerup, Denmark) at 562 nm by spectrophotometry (Spectra Max Plus 384 UV/VIS plate reader, Molecular Devices Inc., Sunnyvale, CA, USA) according to the manufacturer's protocol. Purified protein (25 *μ*g) diluted with nuclease-free water and sample buffer (4x Laemmli Sample Buffer, Bio-Rad Laboratories, Copenhagen, Denmark) was heated at 70°C for 10 min, loaded on precast polyacrylamide gels (Any KD Criterion TGX gel, Bio-Rad Laboratories, Copenhagen, Denmark), and run in duplicate at 200 volts in 1x Tris/glycine/SDS running buffer (25 mM Tris, 192 mM glycine, 0.1% SDS, pH 8.3) (Bio-Rad Laboratories, Copenhagen, Denmark). A protein size standard (MagicMark™ XP, Life Technologies Europe BV, Naerum, Denmark) and an internal calibrator were included on each gel, and positive and negative controls were included in each run. Gel to membrane transfer of protein was performed in a Trans-Blot® Turbo™ Transfer System (Bio-Rad Laboratories, Copenhagen, Denmark) using a LF PVDF Transfer Kit (Bio-Rad Laboratories, Copenhagen, Denmark) according to the manufacturer's protocol prior to 1 hblocking in 2% blocking solution containing Amersham™ ECL Advance™ Blocking Reagent (Fisher Scientific, Roskilde, Denmark) in PBS-T wash buffer (1x PBS and 0.1% Tween 20). Following blocking, membranes were incubated with primary antibody (Table 2) in the blocking solution at 4°C overnight, washed in PBS-T wash buffer, and incubated with goat anti-rabbit secondary antibody (#170-6515, Bio-Rad Laboratories, Copenhagen, Denmark) in the blocking solution for 1 h at room temperature, followed by washing in PBS-T wash buffer. Imaging was achieved by enhanced chemiluminescence (WesternBright Quantum Chemiluminescent HRP Substrate, Advansta Corporation, Menlo Park, CA, USA) in an Odyssey Fc Imager (LI-COR® Biosciences UK Ltd., Cambridge, UK). Optical density of protein bands was determined using the Image Studio™ Software (LI-COR Biosciences UK Ltd., Cambridge, UK). Signal density was normalised to the corresponding actin signal and to the internal calibrator; results were reported as a mean value of the two duplicates. Specificity of the primary antibodies was confirmed by a preabsorption test with the corresponding blocking peptides (Table 2).

### 2.7. Levels of Lipid Oxidation Products

Oxidative damage to brain lipids was assessed by measuring levels of malondialdehyde (MDA) in brain tissue and plasma as described elsewhere [29] and plasma levels of 8-F_2_-isoprostanes (8-ISO) as described by the assay kit manufacturer (cat. number 516351, Cayman Chemical Company, Ann Arbor, MI, USA). This was measured in homogenates of left hemisphere with left half of the cerebellum.

### 2.8. Plasma Levels of Leptin

Plasma levels of leptin were measured using a commercially available luminescent oxygen channelling assay for detection of mouse leptin (cat. number AL521 C/F, Perkin Elmer Inc., Waltham, MA, USA) as described by the assay kit manufacturer, except for the following modifications: rat plasma was treated with activated decolorizing carbon and was then used to prepare standards and controls for the assay. It was furthermore used to dilute samples (4x). The lower limit of quantification (LLOQ) of the assay was 1.2 ng/ml. Sample volume used was 5 *μ*l, and they were analysed as single-determinations.

### 2.9. Statistical Analysis

Data for body weight, food consumption, and whole blood glucose levels was compared by multiple *t*-tests using the Holm-Sidak method; each time point was analysed separately without assuming a consistent SD. For body weight and food consumption, each sex was analysed separately. All other data was evaluated at each time point separately using a two-way ANOVA testing for effect of HI-dosing and sex. In case of interaction, a post hoc Sidak's test for effect of HI-dosing and for effect of sex, respectively, was performed. Second, an additional analysis was performed for parameters measured at both Day 29 and Day 57 of infusion, if there was no interaction or effects of sex and HI-infusion. The CTRL-M and CTRL-F as well as HI-M and HI-F groups, respectively, were pooled together for both time points, and a two-way ANOVA was performed on this data (that is, HI-M+F versus CTRL-M+F) from both time points to test for effect of duration of infusion and for an overall effect of HI-dosing regardless of duration of infusion. In case the first analysis on each time point separately revealed an effect of sex, this second analysis was performed for each sex separately.

## 3. Results

### 3.1. Animals

After surgery, the animals generally behaved normally without any signs of discomfort or stress resulting from the catheter and/or harness. Out of the 152 animals in total, 34 were terminated or died prematurely during the study. Of these, 12 were related to hypoglycaemia, 15 to problems with the infusion system (e.g., catheter becoming disconnected or regressing under the skin), 5 due to poor clinical condition or limb problems, and 2 (controls) for unknown reasons.

**Figure 1:**
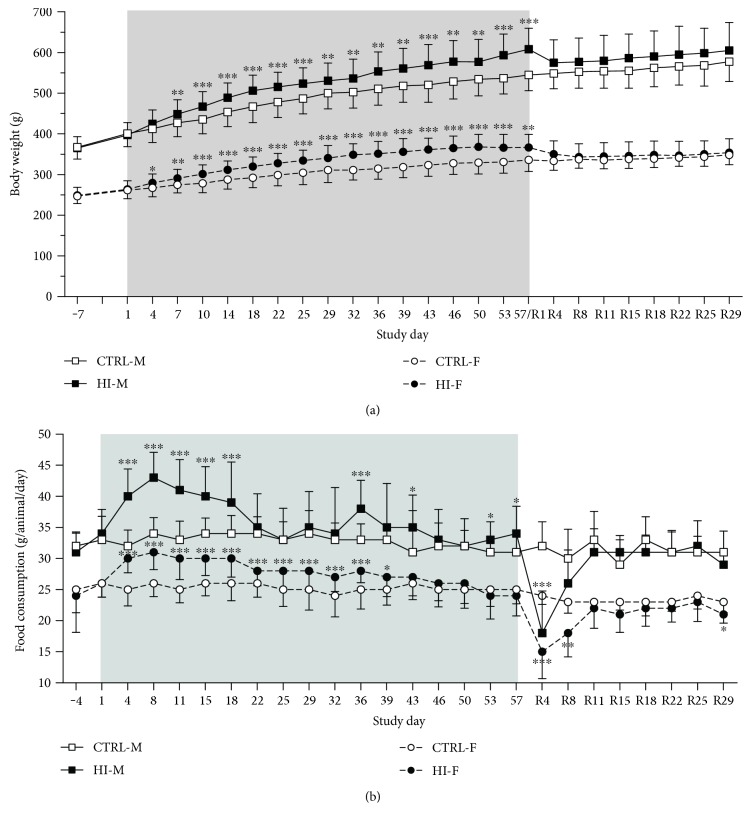
(a) Body weight, group mean + SD. (b) Food consumption, group mean + SD. Grey areas indicate infusion period. Days −7 to 29: *n* = 32 − 38/group per time point, days 32–57: *n* = 20 − 28/group per time point (except for days 53 and 57 for group HI-M, where *n* = 15 − 17/time point), and days R4–R29: *n* = 5 − 10/group per time point. ^∗^*p* < 0.05, ^∗∗^*p* < 0.01, and ^∗∗∗^*p* < 0.001 versus controls.

Mean body weight was significantly increased by approximately 10% after eight weeks of HI-infusion in males and females compared to that of the control groups (Figure 1(a); *p* < 0.001 in HI-M and *p* < 0.01 in HI-F versus CTRL). Following discontinuation of infusion, an initial decrease in body weight was observed in the male and female HI-infused groups, rendering body weights similar to CTRL group body weights for the remainder of the study. Food consumption was generally significantly increased during HI-infusion (Figure 1(b)), with the effect being most pronounced during the first three weeks of HI-infusion (≤26% increase in the HI-M and ≤20% in the HI-F group compared to that in the controls). Discontinuation of infusion was followed by a significant decrease in food consumption in groups HI-M and HI-F by approximately 40% compared to that in the controls during the first four days of the infusion-free recovery period (Figure 1(b); *p* < 0.001). Food consumption was still decreased by Day R8 by approximately 10% in group HI-M (*p* = 0.16) and 20% in group HI-F (*p* < 0.01). There was no significant difference in food consumption from Day R11 and onwards.

### 3.2. Plasma HI Levels and Toxicokinetic Analysis

All the sampled animals from the HI-F and HI-M groups were systemically exposed to HI, and in all plasma samples from the animals in the control groups, HI concentrations were below the LLOQ (25 pmol/L) (data not shown). On Day 1, there was a trend for increasing plasma HI concentrations from 0.5 hours after infusion start. The mean group steady state HI concentrations, *C*_ss_, ranged between 1600 and 3100 pmol/l (Table 3). Despite different dose levels of HI, there was no statistical difference in exposure between mean *C*_ss_ on Day 1 versus Day 53 or between HI-infused males and females.

### 3.3. Blood Glucose Levels

#### 3.3.1. Plasma Glucose Levels

**Figure 2:**
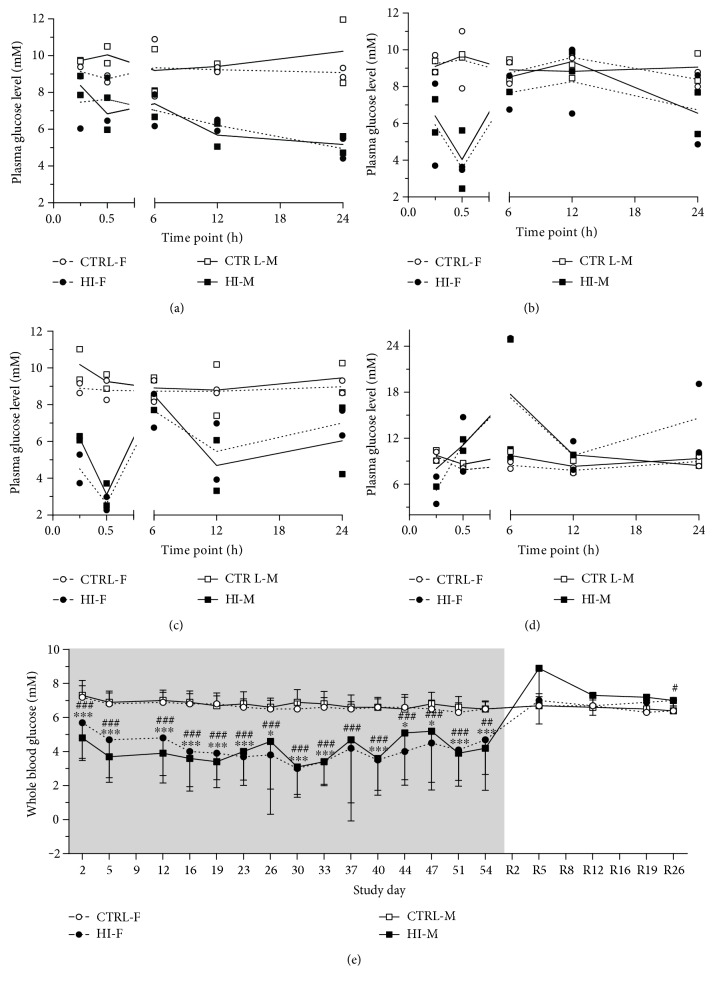
Blood glucose levels. Plasma profile Day 1 (a), Day 25 (b), Day 53 (c), and Day R1 (d). Individual (symbols) and mean (lines) values, *n* = 1 − 2/time point. Infusion start was defined as time point zero on day 1. Time point zero on day R1 corresponds to the time point when infusion was stopped. (e) Whole blood mean values + SD. Grey area indicates infusion period. During infusion: days 2–23: *n* = 28 − 38/group per time point, days 26–51: *n* = 22 − 28/group per time point, and day 54: *n* = 12 − 17/group per time point. During infusion-free period (recovery): *n* = 5 − 10/group per time point. ^∗^*p* < 0.05, ^∗∗^*p* < 0.01, and ^∗∗∗^*p* < 0.001 for HI-M versus CTRL-M; ^#^*p* < 0.05, ^##^*p* < 0.01, and ^###^*p* < 0.001 for HI-F versus CTRL-F. Levels in the HI-M and HI-F groups were only significantly different on two time points: day 5 (*p* < 0.05) and day R12 (*p* < 0.05).

On Day 1, plasma glucose concentrations in the HI-infused males and females groups began to decline steadily from 0.5 h after infusion start, and at the 24 h time point day 1, the values were approximately 60% of the control values (Figure 2(a)). On Day 25 and 53, plasma glucose concentrations were generally lower in the HI-infused groups compared to those in the controls except for two of the time points on Day 25 (6 and 12 h) and Day 53 at 6 h, where levels were similar to those in the controls (Figures 2(b) and 2(c)). Also, for the single time-point measurements on Day 8 and 29 (Table 4), plasma glucose levels in the HI-infused groups were significantly lowered to approximately 50–70% of the control values (*p* < 0.001), with no difference between males and females. From 0.5 to 6 h after termination of infusion on Day R1, plasma glucose levels in groups HI-M and HI-F were generally higher than those in the controls (Figure 2(d)). On Day R2 (single time-point measurements), group HI-M had significantly higher plasma glucose levels than groups CTRL-M (+114%) and HI-F (Table 4, *p* < 0.01 and *p* < 0.05, resp.), whereas on Day R29, plasma levels of MDA on groups.

#### 3.3.2. Whole Blood Glucose Levels

During the infusion period, whole blood glucose concentrations were generally significantly lowered to approximately 4-5 mM in the HI-infused animals compared with stable concentrations of about 7 mM in the control groups (Figure 2(e)), generally with no difference between sexes. After termination of infusion, mean whole blood glucose level in the HI-M group tended to be higher than that in the control animals on Day R5 and Day R12 (Figure 2(e)); however, this was not significant (*p* = 0.055 and *p* = 0.051, resp.). In the remaining infusion-free period, there was no significant difference in whole blood glucose levels between the groups, except for the HI-F group having slightly increased levels on Day R26 (*p* < 0.05).

### 3.4. Protein Levels of Substrate Transporters in Brain Tissue

#### 3.4.1. Effects during Infusion

**Figure 3:**
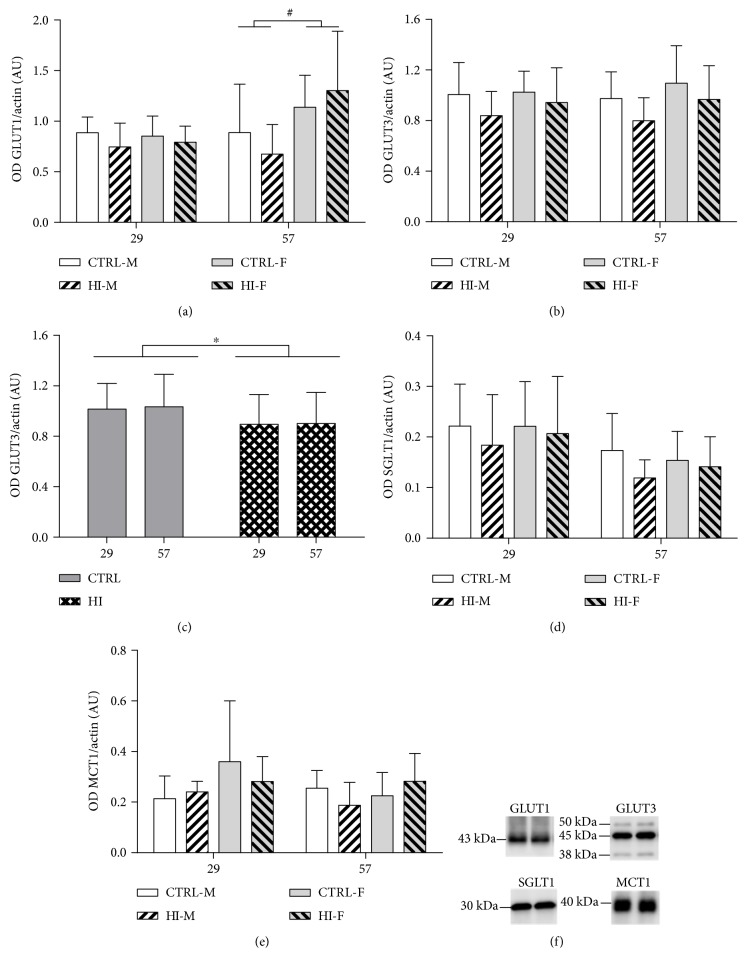
Relative protein levels of substrate transporters at the two different time points during infusion. Mean + SD. (a) GLUT1, each day depicted separately, (b) GLUT3, each day depicted separately, (c) GLUT3, overall effect of HI-dosing, sex pooled. (d) SGLT1 and (e) MCT1, each day depicted separately. (f) Representative pictures of the western blots. CTRL-M: *n* = 7 − 10, HI-M: *n* = 4 − 8, CTRL-F: *n* = 6 − 9, and HI-F: *n* = 6 − 10. OD: optical density, AU: arbitrary units. Actin levels were used as an internal reference. ^∗^*p* < 0.05 for effect of treatment; ^#^*p* < 0.05 for effect of sex.

Only levels of the 45 kDa (astrocytic) isotype of GLUT1 were detected and quantified. Mean levels of GLUT1 protein in brain tissue homogenates were similar for the HI-infused and control groups after 4 weeks (Day 29, *p* = 0.1375) and 8 weeks (Day 57, *p* = 0.8924) of infusion (Figure 3(a)). On Day 57, there was an effect of sex with higher GLUT1 protein levels in females (*p* = 0.0172). Therefore, each sex was tested separately for an overall effect of HI-infusion, revealing no significant effect in males or females (*p* = 0.1655 and *p* = 0.4125).

There was no effect of HI-infusion on GLUT3 levels after 4 weeks or 8 weeks (*p* = 0.1361 and *p* = 0.1140, resp.) (Figure 3(b)). As there was no effect of sex on each time point (*p* = 0.4493 and *p* = 0.1309, resp.), males and females were pooled within each group to test for an overall effect of HI-infusion regardless of duration. This revealed an overall significant decrease (*p* = 0.0389) of GLUT3 levels in the brains from the HI-infused animals (Figure 3(c)).

SGLT1 protein levels were not affected by HI-infusion for 4 or 8 weeks (*p* = 0.4694 and *p* = 0.0703, resp.), although approaching a significant decrease after 8 weeks, and were not different between males and females (*p* = 0.7508 and *p* = 0.7486) (Figure 3(d)). Testing for an overall effect of HI-infusion revealed no significant differences (*p* = 0.1481). MCT1 protein levels were not affected by sex (*p* = 0.0849) or HI-infusion (*p* = 0.6219) after 4 weeks; however, after 8 weeks of infusion, there was interaction between effect of HI-infusion and sex (*p* = 0.0433) (Figure 3(e)). Post hoc testing for effect of HI-dosing in each sex separately and effect of sex in each dose-group separately revealed no significant changes (*p* values all above 0.05). Representative pictures of the western blots are included in Figure 3(f).

#### 3.4.2. Effects after Infusion

There were no effects of HI-dosing or sex on protein levels of any of the transporters after 4 weeks of infusion-free recovery period (data not shown).

### 3.5. Levels of Lipid Peroxidation Products

**Figure 4:**
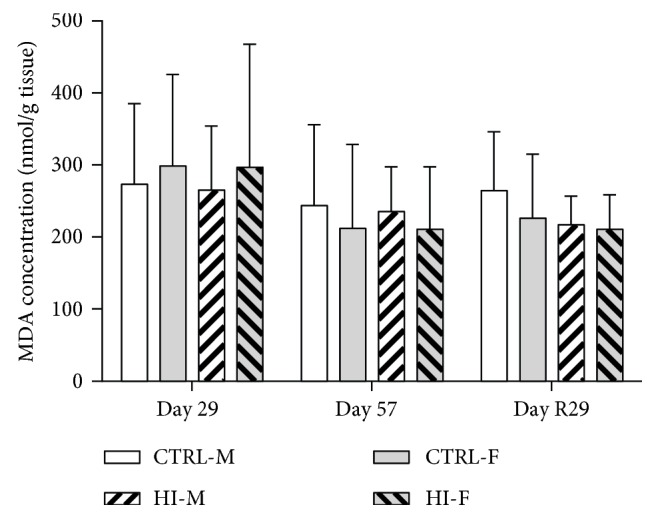
MDA concentration in brain tissue, mean + SD. CTRL-M: *n* = 7 − 10, HI-M: *n* = 5 − 7, CTRL-F: *n* = 8 − 9, and HI-F: *n* = 6 − 9. Statistical analysis showed no effect of HI-dosing or sex at any of the time points.

**Figure 5:**
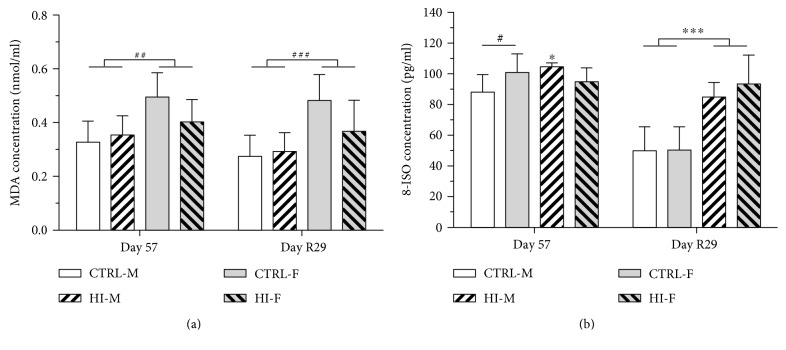
Plasma levels of lipoperoxidation products, mean + SD. (a) MDA, day 56: *n* = 4 − 10 and day R29: *n* = 5 − 10. (b) 8-ISO, day 56: *n* = 4 − 10 and day R29: *n* = 5 − 10. ^∗^*p* < 0.05 and ^∗∗∗^*p* < 0.001 for effect of HI-dosing. ^#^*p* < 0.05, ^##^*p* < 0.01, and ^###^*p* < 0.001 for effect of sex.

Levels of MDA in brain tissue were unaffected by HI-infusion or sex (all *p* values > 0.05) at any of the time points and also in the second test for overall effect of HI-infusion (*p* = 0.5435) (Figure 4). For plasma levels of MDA on Day 57, a two-way ANOVA testing for effect of HI-dosing and sex showed no interaction (*p* = 0.0818), no effect of dosing (*p* = 0.3262), but an effect of sex (*p* = 0.003), with higher levels in females. Day R29: *n* = 5 − 10, a two-way ANOVA testing for effect of HI-dosing and sex showed no interaction (*p* = 0.0639), no effect of dosing (*p* = 0.1704), but an effect of sex (*p* = 0.0003), with higher levels in females (Figure 5(a)). For plasma 8-ISO levels on Day 57, there was interaction (*p* = 0.0129) between effect of HI-infusion and sex. The post hoc analysis revealed an effect of HI-infusion in males (Figure 5(b), *p* = 0.0316), with no effect in females (*p* = 0.4830) and an effect of sex within the CTRL group, with higher levels in females (*p* = 0.0297). On Day R29, 8-ISO levels were significantly increased in the animals previously infused with HI compared to those in the control animals (Figure 5(b), *p* < 0.001).

### 3.6. Plasma Leptin Levels

**Figure 6:**
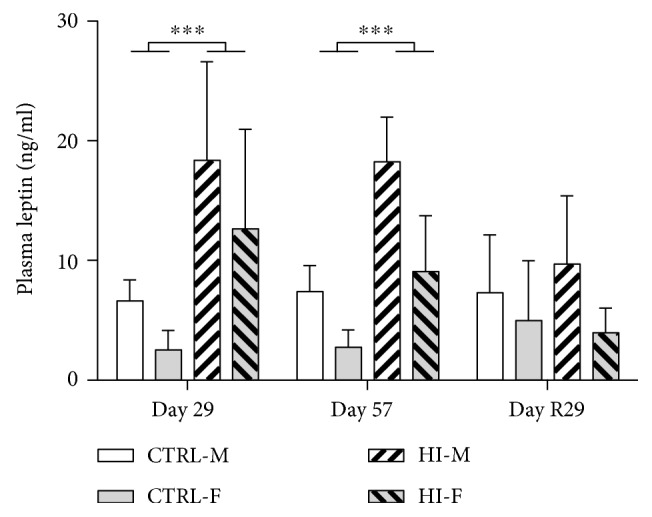
Plasma leptin levels, mean + SD. CTRL-M: *n* = 7 − 10, HI-M: *n* = 4 − 8, CTRL-F: *n* = 8 − 10, and HI-F: *n* = 6 − 9. At all three time points, males had significantly higher leptin levels compared to females (*p* = 0.0325, *p* < 0.0001, and *p* = 0.0182). ^∗∗∗^*p* < 0.001.

Results are shown in Figure 6. HI-infusion caused significant increase of leptin levels after both 28 and 56 days of infusion (*p* < 0.0001 on both days), which was back to normal after 28 days of infusion-free recovery (*p* = 0.6726). At all the three time points measured, leptin levels were significantly higher in males compared to those in females (*p* = 0.0325, *p* < 0.0001, and *p* = 0.0182, resp.).

## 4. Discussion

Interestingly, the present study shows that chronic hyperinsulinaemic hypoglycaemia in healthy rats leads to hyperleptinaemia and a decrease in levels of the neuronal glucose transporter GLUT3 in the brain. Despite the hypoglycaemia and this decrease in glucose transporter levels, no general increase in lipid oxidation production was seen in the brain. Discontinuation of insulin infusion resulted in transient hyperglycaemia and significantly increased plasma levels of lipid oxidation products four weeks later, suggesting systemic lipid oxidation. HI-infusion induced continuous moderate lowering of blood glucose levels to approximately 50–70% of the controls (mean levels of 4–6 mM versus 7–9 mM in the controls), which is more pronounced than the reduction seen after short-term fasting in rats (70–80% of controls) [30–32]. More severe hypoglycaemia (such as levels < 2.5 *mM*, that is, <40% of the controls) leading to clinical signs requiring glucose intervention and euthanasia was observed in several animals within the first few weeks of the study, and consequently, HI doses were lowered. HI-infusion resulted in increased food consumption and increased body weights, a well-known phenomenon of insulin dosing [21, 33–35]. The increase in food consumption seemed most pronounced during the first three weeks of HI-infusion; hereafter, the effect on food consumption attenuated over time. This could be due to a decrease in the counterregulation to hypoglycaemia and hypoglycaemia unawareness, that is, the animals become less responsive to the hypoglycaemia [5], which also explains why plasma glucose levels were lower on Day 29 compared to those on Day 8, despite the dose reduction after approximately two weeks of infusion. HI-infusion was furthermore accompanied by increased levels of circulating leptin by approximately 150–180% in males and 230–400% in females. This hyperleptinaemia was most likely caused by the increased body weights induced by the hyperphagia and hyperinsulinaemia, as circulating levels of leptin are correlated with body fat mass [36].

Relative protein levels of brain glucose transporters GLUT1 and GLUT3 were investigated as they represent the main glucose transporters responsible for the basal glucose transport into astrocytes and neurons, respectively [37]. The neuronal glucose transporter, SGLT1, was also included, as it has very high affinity for glucose, and might therefore play an important role during prolonged periods of persistent hypoglycaemia [7, 38, 39]. Brain tissue levels of the astrocytic glucose transporter GLUT1 were unchanged by insulin-induced hypoglycaemia, in line with other studies with hypoglycaemia of shorter duration (8–12 days) [14, 37]. However, an overall decrease of brain GLUT3 levels was seen in the HI-infused groups versus the controls (decreased by 8–12% in HI-F and 17-18% in HI-M versus the respective control groups). Based on findings in previous studies by others showing that acute hypoglycaemia of 4–7 days causes increased GLUT3 expression, and unchanged GLUT3 levels after a more long-term hypoglycaemic state (8–12 days) [11, 13, 37], any change to GLUT3 levels was expected to be an increase to compensate for the low blood glucose levels. The decrease in cerebral GLUT3 protein levels in the animals in the present study concomitant with high levels of circulating insulin and leptin is in agreement with results from a recent paper showing that neonatal overnutrition in mice causes increased adult body weight, hyperleptinaemia, hyperinsulinaemia, and decreased GLUT3 levels in the hypothalamus [40]. This was further supported by an additional in vivo study, where chronic intracerebroventricular leptin treatment in mice also induced a decrease in hypothalamic GLUT3 levels, with no change to GLUT1 levels [40]. Similarly, others have recently shown that, in diet-induced obese rats with increased plasma levels of insulin and leptin, GLUT3 mRNA levels are decreased in the olfactory mucosa, with no change in GLUT1 mRNA level, compared to those in lean controls [41]. This suggests that leptin levels can regulate brain GLUT3 levels and that they are inversely correlated with hyperleptinaemia causing a decrease in GLUT3 levels. This is supported by in vitro studies as well. A fetal rat hypothalamic neuronal cell line has decreased GLUT3 levels after incubation with leptin [40], and a study with a human neuronal cell line suggests that both insulin and leptin decrease neuronal GLUT3 protein level independently of each other [42]. This downregulation is also in line with the fact that high plasma insulin and leptin levels are usually present concomitant with hyperglycaemia and high fat mass, respectively, that is, during periods of excess supply of nutrients [36, 43]. Reversely, naturally occurring hypoglycaemia induced by fasting is typically accompanied by a decrease in plasma insulin and leptin levels and increased neuronal GLUT3 levels, presumably to compensate for restricted supply of glucose from peripheral circulation [40]. Therefore, the hyperinsulinaemia and hyperleptinaemia may act in concert to decrease brain GLUT3 levels in the present study, superseding the hypoglycaemic signal to increase or maintain levels.

Total protein levels of the other neuronal glucose transporter, SGLT1, measured in brain tissue were unaltered by HI-infusion for up to 8 weeks. Not much is known about the regulation of SGLT1 levels in the brain, and since this transporter is mainly located intracellularly [38, 44–46], it may potentially serve as a reserve pool ready for recruiting to the plasma membrane when needed. This is supported by in vitro studies showing increased plasma membrane SGLT1 levels in glucose-deprived epithelial cells [47, 48] and an in vivo study reporting an increase in SGLT1-specific glucose uptake in the rat brain, when an epileptic seizure was induced as a model for metabolic changes in the brain [38]. This was hypothesised to be due to increased translation of SGLT1 and/or by translocation of SGLT1 protein from an intracellular location to the plasma membrane [38]. Additionally, regulation of SGLT1-mediated glucose transport through changes in affinity has also been shown in vitro [49, 50]. These results collectively suggest that chronic insulin-induced hypoglycaemia would cause translocation and/or upregulation of SGLT1 levels. However, in the present study, there was a tendency for a decline in SGLT1 levels (6-7% in HI-F and 17–32% in HI-M versus the respective controls), which approached statistical significance after 8 weeks of HI-infusion (2-way ANOVA, *p* = 0.0703) and was significantly decreased in HI-M after 8 weeks, when performing a post hoc unpaired *t*-test on group HI-M versus CTRL-M (*p* = 0.0436). SGLT1 levels have been shown to be decreased by leptin in vitro in rat jejunal mucosa [51], as well as in vivo in the small intestine of an obese type 2 diabetic mouse model with hyperleptinaemia [52]. This suggests that, similar to GLUT3, SGLT1 in the brain may be prone to regulation by leptin with hyperleptinaemia inducing a downregulation. However, this remains to be investigated further.

As mentioned above, HI-infusion caused no change in GLUT1 levels; however, brain GLUT1 levels were significantly higher in females compared to those in males on Day 57 (16-17 weeks of age). In agreement with this, higher GLUT1 mRNA levels have been shown in adipocytes from females compared to those from males in mice [53], seemingly mediated through sex steroids, which is further corroborated by a study in female estradiol-treated rhesus monkeys [54]. The relevance of this sex difference in GLUT1 expression is unknown, and it was not present in 12-13-week-old (day 29) or 20-21-week-old (day R29) female rats in the present study.

An additional astrocytic transporter, monocarboxylate transporter MCT1 [55], was included to investigate if chronic insulin-induced hypoglycaemia would lead to upregulation of this transporter in order to increase transfer of astrocytic lactate formed by breakdown of astrocytic glycogen stores to the extracellular space to make it available as an alternative energy substrate for the neurons. As for GLUT1, protein levels of MCT1 were similarly not changed by chronic hypoglycaemia, which could indicate that MCT1 transporter levels are adequate to meet the demand for lactate transport or the transporter's affinity might be altered towards a prioritisation of lactate transport.

It is very interesting that, during this restricted supply of glucose from peripheral circulation, the animals increase body weights and become hyperleptinaemic. Hyperleptinaemia is typically associated with hyperphagic and/or diet-induced obesity also accompanied by hyperinsulinaemia as its levels are correlated with fat mass [36, 43], as mentioned above, and acts centrally to decrease appetite similar to insulin. Despite high circulating levels of insulin and leptin in the present animal model of hypoglycaemia, the animals have increased food consumption, presumably driven mainly by the hypoglycaemia [33]. Normally, hyperinsulinaemia would only be present with hyperglycaemia, and not much is known about the central response to chronic hyperinsulinaemic hypoglycaemia. The hypoglycaemia-induced hyperphagia despite hyperinsulinaemia has been shown before [21, 34], indicating that, with regard to control of appetite, the glucose availability dominates over insulin and leptin signalling, at least initially in the present model.

Following insulin withdrawal, transient increased blood glucose levels were accompanied by significantly decreased food consumption and body weight loss. Such transient hyperglycaemia after cessation of HI-infusion has been attributed to suppressed endogenous insulin production [56–58]. However, a study has shown that *β*-cells quickly regain insulin production within a few days and is back to normal within 5 days [35]. This is most likely also what these changes reflect in the present study, with hyperglycaemia inducing a counterregulatory decrease in food consumption until about 8–11 days after cessation of HI-infusion, where blood glucose levels are back to control levels.

Quantification of biomarkers of lipid oxidation in the brain and plasma was included in the present study as oxidative stress appears to play an important role in the pathogenesis of neuronal damage caused by insulin-induced hypoglycaemia [7, 17–19]. MDA and 8-ISO are both products of oxidative damage to lipids and are considered biomarkers of lipid oxidation [59, 60]. Levels of MDA and ISO-8 were unaltered during HI-infusion, suggesting that, in contrast to acute and recurrent insulin-induced hypoglycaemia [17, 19, 61], lipid peroxidation is not increased in the brain during chronic blood glucose lowering. Surprisingly, plasma levels of MDA were higher in females compared to those in males, despite an apparent stronger antioxidant defence system in females compared to that in male rats [62]. Interestingly, plasma levels of 8-ISO were significantly increased after a 4-week infusion-free recovery period, which may possibly be due to the transient hyperglycaemia giving rise to oxidative stress [63, 64]; however, this remains to be clarified.

## 5. Conclusions

In conclusion, the present study suggests that the brain of normoglycaemic healthy rats subjected to chronic insulin-infusion is prone to a downregulation of levels of GLUT3, the neuronal glucose transporter, despite a lowering of blood glucose levels by 30–50%. The hyperinsulinaemic hypoglycaemia was, furthermore, accompanied by increased food consumption and body weights as well as hyperleptinaemia. Recent studies by others suggest that the latter may have induced the decrease in GLUT3 level. Despite hypoglycaemia and decreased levels of neuronal glucose transporters, there were no changes to lipid oxidation in brain tissue.

Insulin withdrawal following chronic infusion induced transient hyperglycaemia—most likely a consequence of reduced endogenous insulin production—as well as transiently decreased food consumption and body weight and apparent increased systemic oxidative stress.

## Figures and Tables

**(a):** 

Day 1	Day 29	Day 57	Day R2	Day R29
	Sacrifice	Sacrifice	Sacrifice	Sacrifice
	*n* = 9/*sex*/group	*n* = 10/*sex*/group	*n* = 9/*sex*/group	*n* = 10/*sex*/group
	*Infusion*		*Infusion-free recovery*	

**(b):** 

Group	*n*	HI dose during infusion (nmol/kg/day)
CTRL-M/CTRL-F	*38/38*	0/0
HI-M/HI-F	*38/38*	72>60^*∗*^/54>48^*∗*^

HI: human insulin, CTRL-M: control group males, CTRL-F: control group females, HI-M: HI-infused males, HI-F: HI-infused females. ^∗^Doses were lowered day 12 or day 19 due to clinical signs of severe hypoglycaemia and mortalities related to hypoglycaemia. As the animals started infusion in the two cohorts, doses were lowered on two different dosing days.

**Table 2:** Antibodies used for western blotting.

Transporter	Primary antibody (cat. number)	Company	Working dilution	Size of band(s) detected (kDa)	Blocking peptide (cat. number)	Host species of primary antibody	Secondary antibody dilution^d^
GLUT1	07-1401	MLS^a^	1:8000	43	N/A	Rabbit	1:3000
GLUT3	ab15311	Abcam^b^	1:400	38, 45, and 50	N/A	Rabbit	1:1500
SGLT1	ab14686	Abcam	1:1000	30, (42)	ab190911	Rabbit	1:2000
MCT1	AB3540P	MLS^a^	1:5000	40	N/A	Rabbit	1:4000
Actin	Ab1801	Abcam	1:1000	10, 25, 30, and 42^c^	ab13771	Rabbit	1:2000

N/A, not applicable as the corresponding blocking peptide was not commercially available; instead, one was custom synthesized (CASLO ApS, Kgs. Lyngby, Denmark) according to the peptide sequence supplied by the manufacturer of the primary antibody. All custom-synthesized blocking peptides were supplied at >95% purity. ^a^Merck Life Science A/S, Hellerup, Denmark. ^b^Abcam plc., Cambridge, UK. ^c^Triple band. ^d^The same secondary goat anti-rabbit (#170-6515, Bio-Rad Laboratories, Copenhagen, Denmark) was used for all primary antibodies.

**Table 3:** Estimated group mean *C*_ss_ of HI in plasma (pmol/l).

Group	*n*	Day 1^a^	*(Range)* ^b^	*n*	Day 53	*(Range)* ^b^
HI-M	*6*	1780	*(502–3050)*	*8*	1780	*(222–7870)*
HI-F	*6*	3120	*(135–14900)*	*6*	1590	*(180–4360)*

^a^6–24 h. ^b^Of individual means.

**Table 4:** Single time-point plasma glucose level (mM), mean ± SD.

Group	*n*	Day 8^a^ (difference from control)	*n*	Day 29 (difference from control)	*n*	Day R2 (difference from control)	*n*	Day R29^b^
CTRL-M	*36*	9.20 ± 1.06	*7*	8.57 ± 0.317	*7*	7.52 ± 0.652	*10*	8.47 ± 0.75
HI-M	*35*	6.24 ± 3.19^*∗∗∗*^ (−32%)	*8*	4.30 ± 1.883^*∗∗∗*^ (−50%)	*6*	16.10 ± 7.855^*∗∗*^ (+114%)	*5*	9.83 ± 1.74
CTRL-F	*37*	9.05 ± 0.82	*8*	9.01 ± 0.700	*6*	8.19 ± 1.003	*9*	8.42 ± 0.62
HI-F	*37*	6.15 ± 2.74^*∗∗∗*^ (−32%)	*8*	4.84 ± 1.394^*∗∗∗*^ (−46%)	*6*	9.55 ± 1.401^#^	*9*	8.75 ± 1.04

^a^6 h time point. ^b^0.25 h time point. ^∗∗^*p* < 0.01 and ^∗∗∗^*p* < 0.001 versus control; ^#^*p* < 0.05 versus HI-M.
